# Perspectives on Oncolytic *Salmonella* in Cancer Immunotherapy—A Promising Strategy

**DOI:** 10.3389/fimmu.2021.615930

**Published:** 2021-02-25

**Authors:** Ding Wang, Xiaodong Wei, Dhan V. Kalvakolanu, Baofeng Guo, Ling Zhang

**Affiliations:** ^1^Department of Pathophysiology and Key Laboratory of Pathobiology, Ministry of Education, College of Basic Medical Sciences, Jilin University, Changchun, China; ^2^ Department of Microbiology and Immunology and Greenebaum Comprehensive Cancer Center, School of Medicine, University of Maryland, Baltimore, Baltimore, MD, United States; ^3^Department of Plastic Surgery, China-Japan Union Hospital, Jilin University, Changchun, China

**Keywords:** *Salmonella*, cancer immunotherapy, tumor microenvironment, combination therapy, bacterial therapy

## Abstract

Since the first reported spontaneous regression of tumors in patients with *streptococcus* infection, cancer biological therapy was born and it evolved into today’s immunotherapy over the last century. Although the original strategy was unable to impart maximal therapeutic benefit at the beginning, it laid the foundations for the development of immune checkpoint blockade and CAR-T which are currently used for cancer treatment in the clinics. However, clinical applications have shown that current cancer immunotherapy can cause a series of adverse reactions and are captious for patients with preexisting autoimmune disorders. *Salmonellae* was first reported to exert antitumor effect in 1935. Until now, numerous studies have proved its potency as an antitumor agent in the near future. In this review, we summarize the currently available data on the antitumor effects of *Salmonella*, and discussed a possibility of integrating *Salmonella* into cancer immunotherapy to overcome current obstacles.

## Introduction

Using bacteria to treat tumors has long been a controversial subject. Dr. William B. Coley, a surgeon at the New York Hospital, first reported the spontaneous regression of tumors in patients with *streptococcus* infection at the end of the 19th century (1). In the later 40 years, Coley concentrated on applying the “Coley’s Toxins”, a variety of antitumor mixture of heat-killed *Streptococcus pyogenes* and *Serratia marcescens*, to patients with sarcomas, carcinomas, lymphomas, melanomas, and myelomas (2, 3). By simulating symptoms of infection without the risks of bacteremia, “Coley’s Toxins” account for complete tumor regression in many cases (2, 3). Subsequently, *Salmonella* was first observed a tumor therapeutic effect on animal models from a hemorrhagic allergy experiment reported in 1935 (4). However, because of the concerns about non-reproducible, uncertain, and unpredictable effects in the early trials, the development of chemoradiotherapy and surgical treatment soon rose to prominence and took a dominant position in the following decades for tumor therapy (3). The success of *Bacillus Calmette Guerin* (BCG) against superficial bladder cancer once again brought attention to the treatment of tumors with bacteria (5, 6). With the development of molecular biology, comprehensive sequencing of bacterial genomes and abundance of gene construction methods have greatly enhanced the plasticity of bacteria as antitumor agents (7). Currently, the antitumor effects of *Salmonella* have been widely studied pre-clinically, in which *Salmonella* can target and colonize into tumor tissues, directly kill tumor cells, and cause changes in tumor immune microenvironment to enhance host tumor recognition and elimination (8, 9). Nevertheless, clinical studies showed that the robust antitumor effects of *Salmonella* have not been fully exhibited in human patients (8), which raises a further claim to the exploration of *Salmonella* modification, as well as augment other treatment strategies.

Presently, remarkable clinical achievements of immune checkpoint blockade and chimeric antigen receptor T cell therapies (CAR-T) enable cancer immunotherapy as another important antitumor strategy after chemoradiotherapy and surgical treatment (10). However, clinical studies in recent years have also revealed the side effects, cancer heterogeneity and limitations of patient selection in cancer immunotherapy (1, 11). In order to expand the applications, obtain durable therapeutic responses and reduce related adverse events of cancer immunotherapy, more precise treatment, complex combinations of immunomodulatory agents, and further activation of host immune system against tumor cells become the future research direction.

In this review, we summarize the present research on the antitumor effects of *Salmonella*, and describe the development and limitations of current cancer immunotherapy. We will also consider the advantages of *Salmonella* in tumor targeting, immune regulation and engineering plasticity. In conclusion, *Salmonella* is expected to further enhance the consistency, durability and effectiveness of cancer immunotherapy.

## *Salmonella* in Cancer Therapy

**Figure 1:**
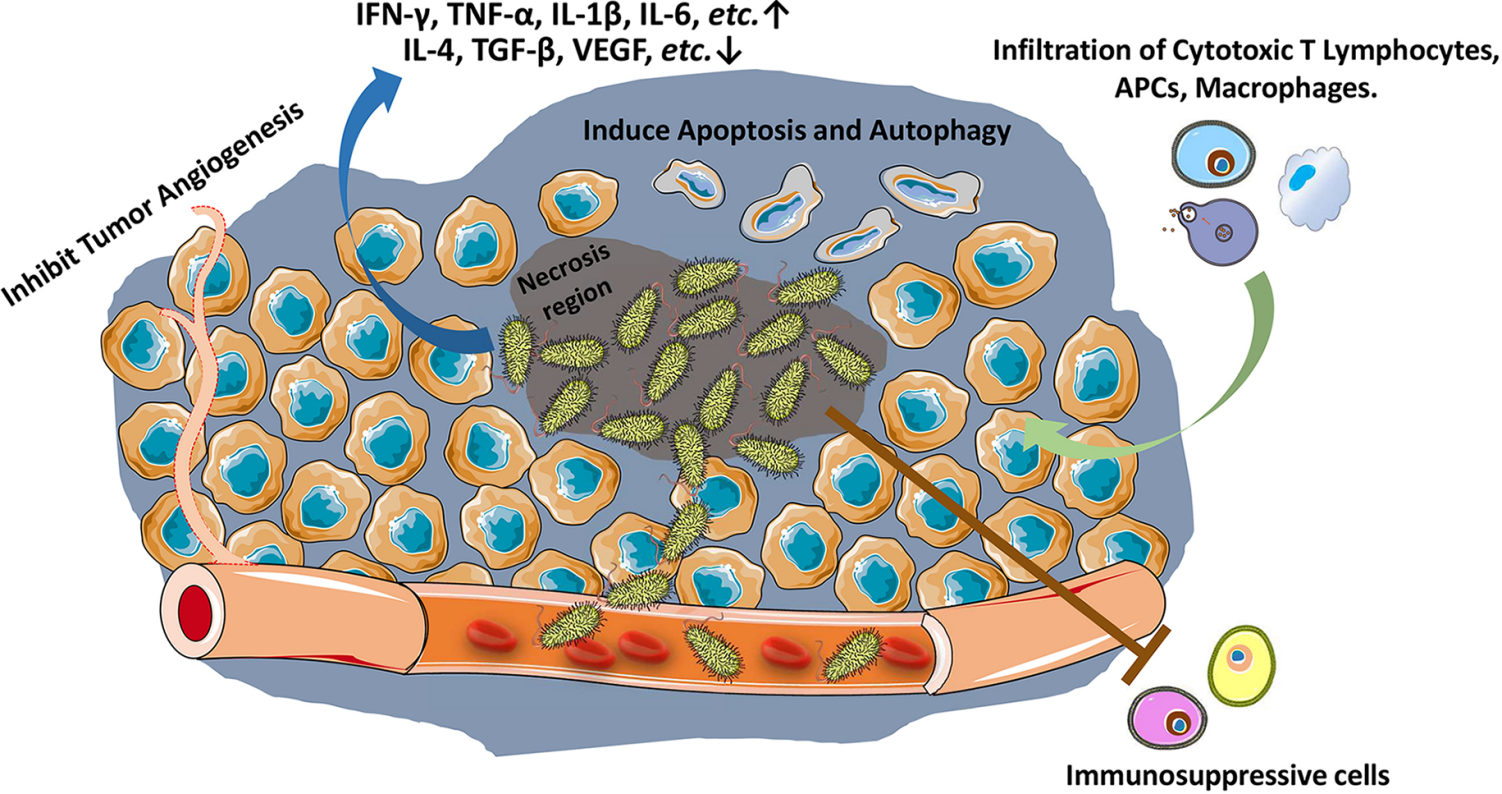
*Salmonella* stimulates host immune response against tumors. *Salmonella* accumulates in tumors (especially in necrosis region), inhibits tumor angiogenesis, and induces apoptosis and autophagy in tumor cells. *Salmonella* increases and activates cytotoxic T lymphocytes, antigen presenting cells (APCs) and macrophages against tumor cells, reduces tumor infiltration of Treg cells, and ablates the immunosuppressive capacity of myeloid-derived suppressor cells (MDSCs) and Tumor-associated macrophages.

Many bacteria have been found the capacity to colonize into tumor microenvironment (TME) (12). Among them, *Salmonella typhi* first showed a significant tumor therapeutic effect on sarcoma in mouse model (4). The antitumor effect of *Salmonella* is mainly exerted through bacterial-intrinsic and host immune mechanisms. The attenuation of bacteria, which reduces the bacterial toxicity owing to the secretory factors, may diminish the intrinsic oncolytic activity of bacteria to a certain extent (12, 13). Such loss of intrinsic antitumor capacity may also augment the other capacity of *Salmonella*, *e.g.*, host immune response against tumors (14). As early as the 19th century, it was discovered that bacteria and their products stimulate the host immune system for inhibiting tumor growth (15, 16). Since the first report about *Salmonella* inhibiting solid tumor growth in 1930s (4), numerous studies have confirmed the ability of *Salmonella* to stimulate immune system against cancer (**Figure 1**) (3, 8). Both innate (17, 18) and adaptive immune responses are augmented (19–21).

### Tumor Targeting Capacity

**Figure 2:**
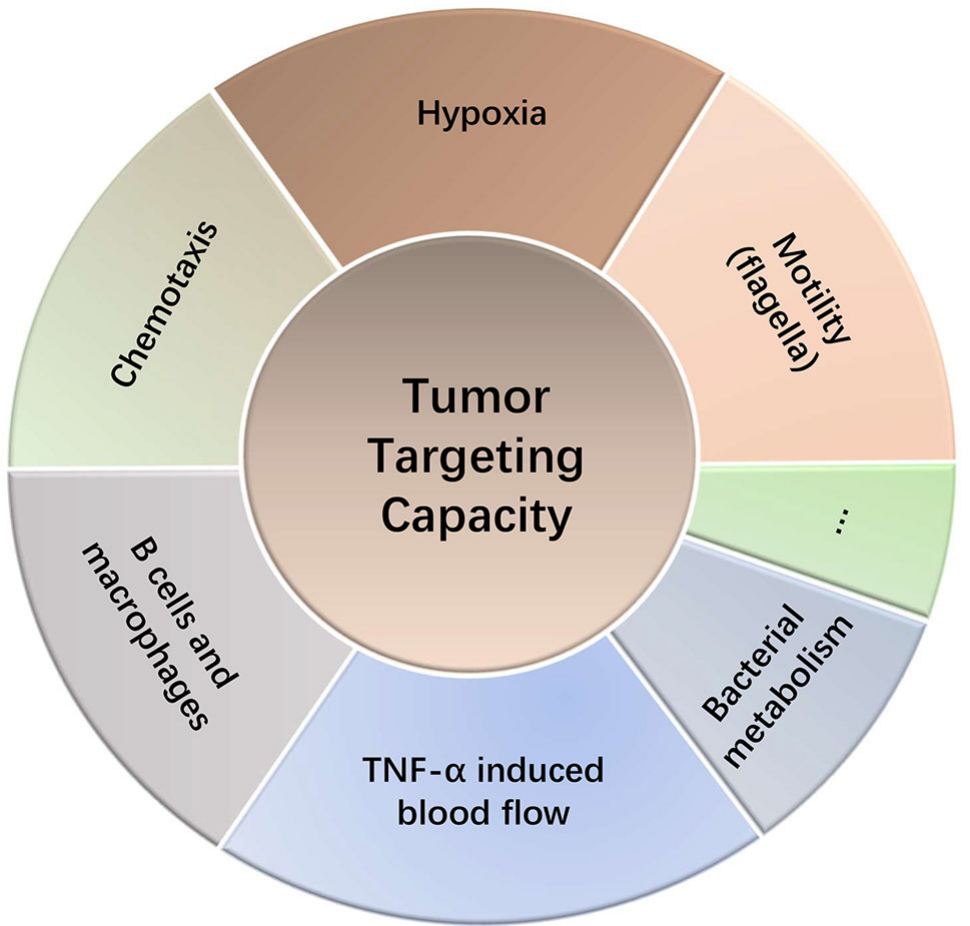
Tumor targeting capacity of *Salmonella*.

*Salmonella* has been shown enrich in the tumors > 1,000-fold over in normal tissues and cause robust antitumor effects in animal models (8). Upon administration into animals, the initial levels of *Salmonella* between tumors and other tissues were not significantly different. *Salmonellae* in the circulatory system and other tissues were cleared within hours to days, while *Salmonella* that entered tumors took footing, colonized and grew (22). The tumor-homing ability of bacteria is probably related to the unique immunosuppressive and biochemical environment of tumors (23). Although tumor-targeting mechanism of *Salmonella* is still not completely understood, it might be related to the following aspects (**Figure 2**):

Hypoxia in tumors permits bacterial colonization of *Salmonella*, a facultative anaerobe (8). *Salmonellae* in tumors migrate away from vasculature and prefer to thrive in the necrotic zones (24). This is also partly due to the availability of nutrients in the necrotic area for bacteria and concealment from the immune system (25). Studies involving both anaerobe and facultative anaerobe showed that the intravenously injected bacteria did not colonize in other non-tumor tissues with hypoxia or inflammatory lesions (26), indicating other potential mechanisms for their tumor homing ability. The genetically modified *Salmonella typhimurium* strain YB1 possessed an enhanced hypoxia selectivity exhibited a stronger tumor-targeting and antitumor ability than the parental strain SL7207 (27). Chemotaxis toward components in TME is another potential mechanism that accounts for the preferential homing into tumors by *Salmonella*. Each chemotactic receptor may distinctly guide *Salmonella* into target tumors, depending on the TME. Kasinskas et al. found that chemotaxis of *Salmonella* is essential for the initial accumulation in tumors, where the quiescent and necrotic cells are crucial for their survival (28). Further research suggests that responsive chemotactic receptors (aspartate, serine and ribose/galactose receptor) can initiate chemotaxis, penetration and direct *Salmonellae* toward necrosis (29). Silva-Valenzuela et al. (30, 70) showed that the chemotactic gene cheY and the motility gene motAB of *Salmonella* are crucial for colonizing tumors. And eutC, an ethanolamine metabolism related gene, also confers a tumor preference of *Salmonella* (30, 31). In addition, motility (flagella) is also necessary for tumor-targeting ability as mutated *Salmonella* fail to colonize the tumors (32). Interestingly, Stritzker et al. (33) arrived at an opposite conclusion. Their *in vivo* studies showed that chemotaxis and motility of *Salmonella* have no impact on tumor colonization. They suggest that the tumor-trafficking ability may be related to a passive mechanism by bacterial metabolism and host macrophages (33). Furthermore, studies in B cell-deficient mice showed higher accumulation of *Salmonellae* in tumors and increased tumor growth inhibition. However, B cells are also required for tumor targeting by restricting the dissemination of *Salmonella* to normal tissues (19). *Salmonella* can induce large amounts of blood flow into tumor tissues by increasing the secretion of TNF-α, which in turn leads to more *Salmonella* entering tumor tissues (34). The tumor targeting ability also contributes to another application of *Salmonella*. Panteli et al. (35) created an engineered fluorescein-secreting *Salmonella* to detect small tumors and metastases. As a nest for *Salmonella* accumulation, tumors and metastases constantly export fluorescent biomarker for detection (35). Similarly, Xiong et al. administrated *Salmonella* carrying a targeting peptide ubiquicidin labeled with fluorescent dye into mouse models, which detected using the multimodal imaging of tumors (36).

### Intrinsic Oncolytic Activity

Similar to viruses, *Salmonella* kills tumor cells using its intrinsic oncolytic activity (37). Active division of tumor cells and the necrosis inside tumors provide a conducive environment for *Salmonellae* (25). *Salmonella* accumulating in tumors deprive extracellular nutrients from tumor cells (38), which promotes apoptosis in tumor cells (39). *Salmonella* influxes into solid tumors up-regulates the production and release of TNF-α, which leads to tumor hemorrhage and *Salmonella* invasion, and eventually inhibits tumor angiogenesis (34). The vessel destruction by *Salmonella* would be augmented by high tumor vascularity (40). Subsequent studies showed that *Salmonella* can also inhibit tumor angiogenesis through AKT/mTOR pathway by suppressing the levels of HIF-1α and VEGF (41). Besides, this anti-angiogenic capacity can be boosted by *Salmonella* with engineering or a combination with Triptolid (42). In addition to destroying the microenvironment required for tumor growth, *Salmonella* can also kill tumor cells by secreting or inducing antitumor agents. As the pathogen-associated molecular patterns (PAMPs) of *Salmonella*, LPS and flagella have been shown antitumor activities in many studies, whose elimination accounts for a loss of therapeutic potency (43–47). LPS from *Salmonella* can increase TNF-α and the tumor specific response of CD8^+^ T cells (45). While flagellin from *Salmonella* can reduce the amount of regulatory T cells (T_reg_) in TME (43). When flagellin affects tumors, TLR4 and TLR5 were shown to play important role in this process using mouse models (43, 44). An engineered flagellum of *Salmonella* has been shown to exert strong antitumor effect (47). *Salmonella* can also inhibit tumors through producing NO in TME, where nitrate reductase secreted by *Salmonella* converts nitrates and nitrites into NO (48, 49). Autophagy is a defensive mechanism of host cells against infection and intracellular proliferation of pathogens (50). Blockade of *Salmonella*-induced autophagy was shown improves cytotoxicity of *Salmonella*, and increases tumor cells apoptosis (51). Lee et al. showed *Salmonella* induce melanoma cell autophagy by restraining AKT/mTOR pathway (52). However, this study indicated that autophagy might also a pattern for *Salmonella* induced cell death. They showed that, 3-Methyladenine, an autophagy inhibitor, suppressed the antitumor effect of *Salmonella* (52). Previous study also found that SipB, a *Salmonella* protein with membrane fusion activity, causes macrophage cell death by inducing autophagy (53). Hence, the question whether autophagy contributes to or blocks the cell death induced by *Salmonella* needs to be confirmed by further research. Another advantage with *Salmonella* therapy is its preference for metastases. Monotherapy with *Salmonella* suppressed tumor growth and metastasis in nude mice (24). Further investigations showed that *Salmonella* downregulated the AKT/mTOR pathway in tumor cells and suppressed the expression of matrix metalloprotease MMP9 (54).

### Induction of Inflammation

*Salmonella* induces proinflammatory responses which are critical for its antitumor effects. Similar to *Salmonella*, *E. coli* also accumulates in tumors, but does not induce antitumor effects. The reason might be that *E. coli* colonization in tumors does not induce a same inflammatory response as *Salmonella* (55). Chirullo et al. reported that, after *Salmonella* administration, the number of monocytes/macrophages (F4/80^+^-Ly6C^+^) and CD8^+^ T cells was significantly up-regulated (56) in the spleens of mice. The expression of IFN-γ and IL-1β was also up-regulated in tumors after *Salmonella* administration (18, 19, 21). Calreticulin, a protein related to immunogenic cell death pathway, is increased in tumor tissues after *Salmonella* infection (56). Likewise, Lee et al. (18) showed that infiltration of macrophages, neutrophils, and CD8^+^ T cells in mouse tumors was increased after *Salmonella* infection. IFN-γ and IFN-inducible chemokines CXCL9 and CXCL10 are also shown to be up-regulated (18). TLR4 is critical for this response, as the antitumor activity was not mounted in *Salmonella* treated TLR4 defective mice (18). Another study showed that the flagellin of *Salmonella* inhibits the numbers of CD4^+^CD25^+^ T_reg_ cells in TME through TLR5 (43). Besides, B cells have been shown to play an important role in regulating IFN-γ expression and adaptive immunity after *Salmonella* treatment (19, 57). Tumor cells infected with *Salmonella* secrete more TNF-α (58). Furthermore, *Salmonella* also elicits transformation of immunosuppressive myeloid-derived suppressor cells (MDSC) into TNF-α-secreting cells, and reduces the infiltration of T_reg_ cells into tumors (59, 60). Some studies also showed that LPS from *Salmonella* induces systemic or local production of massive TNF-α in leucocytes which activates recruitment of macrophages and neutrophils (45, 61, 62). *Salmonella* infection actives the NF-κB pathway in lymphocytes, leading to an increased secretion of proinflammatory factors (63). Multiplex suspension arrays in melanoma models revealed that, *Salmonella* caused a significant up-regulation of proinflammatory factors, such as IL-6, IL-1α, IL-12p70, IL-17, and IL-13, and chemokines, such as G-CSF, GM-CSF, MIP-1α, MIP-1β and *etc*. in the TME (42). In addition, *Salmonella* up-regulated the expression of iNOS in tumors, which is attributed to intratumoral myeloid cells (17). Studies showed that the rise in IFN-γ and iNOS might play an important role in the increase of neutrophils, activated CD8^+^ T cells and natural killer (NK) cells (17, 18). On the other hand, *Salmonella* also down-regulated the expression of immunosuppressive factors such as ARG-1, IL-4, TGF-β, and VEGF (17, 64). *Salmonella* also activates inflammasome formation in myeloid macrophages, which activates the expression of caspase-1, NLRP3 and IPAF, and induces apoptosis of tumor cells (55). This process might include two distinct effects: 1) *Salmonella* directly stimulates macrophages to secrete proinflammatory factor using its flagellin (65); 2)ATP released from *Salmonella*-damaged tumor cells stimulates macrophages to release inflammasomes (55).

### Effects on Immune Cell Infiltration

*Salmonella* was shown to facilitate the recruitment of both innate and adaptive cells to the tumor (66). Avogadri et al. (2005). observed a large leukocyte infiltration into necrotic tumor areas with a high *Salmonella* accumulation. Initially (first 2 days), the recruited immune cells mainly consisted of granulocytes. T lymphocytes (both CD4^+^ and CD8^+^) and B lymphocytes (CD19^+^) were recruited at later following *Salmonella* injection (67). Other studies also indicate the infiltration of lymphocytes, including CD4/8^+^ T cells and NK cells to the site of tumor after *Salmonella* infection (67, 68). Chirullo et al. showed that *Salmonella* up-regulates calreticulin in TME (56), which may contribute to the Cytotoxic T Lymphocytes (CTLs) infiltration in patients with ovarian and non-small cell cancers (69). Moreover, Clay et al. found that polarization of CD4^+^ T cells in *Salmonella* infected site is regulated by T_reg_ cells (70). *Salmonella* enhances the infiltration of neutrophils into the tumor, which has been demonstrated in many researches (46, 48, 68, 71). Some studies showed the antitumor capacity and tumor antigen-presenting function of neutrophils after the administration of *Salmonella* (72). However, these neutrophil populations are heterogenous, which may exert either positive or negative effects on tumor growth (73). Chen et al. indicated that Triptolide, diterpenoid triepoxide compound purified from *Tripterygium wilfordii*, augments antitumor effect of *Salmonella* by reducing neutrophils infiltration in melanoma (42). Whether the subset of neutrophils recruited by *Salmonella* would exert pro- or antitumor functions is still worth a further investigation. Besides, administration of *Salmonella* also recruits macrophages into tumor tissue, which then secrete TNF-α and IL-1β for exerting anticancer effects (17, 46).

### Sensitization on Tumor Immune Microenvironment

In addition to inducing inflammation, *Salmonella* also promotes T cell-dependent immune responses. Because of the antigenic determinants of *Salmonella* on tumor cell surfaces, it allows to CTLs to kill tumor cells (67). Dendritic Cell (DC) present exogenous antigens to T cells through the cross-presentation pathway. Such presentation of tumor peptides is crucial for anti-tumor T cell development (74). *Salmonella* not only increases the number of active DCs in mouse lymph nodes but also enhances tumor antigen presentation by DCs (59, 75). Saccheri et al. (2010). demonstrated that bacteria-treated melanoma cells can establish functional gap junctions with adjacent DCs. The antigenic peptides from tumor cells can be transferred to DCs through the gap junctions, which then present these peptides to CTLs against tumor cells. During this process, connexin 43 (Cx43), a ubiquitous protein that forms gap junctions and normally lost in melanoma cells, was up-regulated after *Salmonella* administration (75). Besides, *Salmonella* was shown reduce the expression of indoleamine 2, 3-dioxygenase 1 (IDO) *via* inhibiting AKT signaling pathway, which is related to immune tolerance and T cell apoptosis (76). Although *Salmonella* targets tumors, most of them live in the necrotic areas. Neutrophils surrounding the necrotic areas actually prevent *Salmonella* from spreading to non-tumor cells. But when those neutrophils are depleted, *Salmonella* can then breach that barrier and further suppress tumors (77, 78).

The inhibitory effect of tumor immune microenvironment is an important reason for tumor immune-suppression and the poor efficacy of tumor immunotherapy. One study tested if the expression of immune checkpoint PD-L1 was affected by *Salmonella* on a variety of cancer cell lines. Data from these experiments demonstrated that *Salmonella* downregulated PD-L1 expression in a p-AKT, p-mTOR and p-p70S6K involved manner. As a result of reduced apoptosis, the T cell numbers rose (79). *Salmonella* also affects other immunosuppressive cells (60) in the TME. The down-regulation of IDO inhibits the numbers, activation and censorship of T_reg_ cells on tumor immunity (60, 76, 80, 81). Intratumoral injection of attenuated *Salmonella* produces antitumor effect by redirecting activated TNF-α secreting neutrophils to the tumor site and reducing T_reg_ cells in the draining lymph nodes of the tumor (82). Liu et al. found that tumor suppression by *Salmonella* is associated with a down-regulation of CD44^high^ and CD4^+^CD25^+^ T_reg_ cells and the LPS and Braun lipoprotein of *Salmonella* were critical for such response (83). The MDSCs are crucial contributors to tumor progression (84). Study from Kainala et al. (2014). showed *Salmonella* ablates the immunosuppressive capacity of MDSC and Tumor-associated macrophages (17). Chang et al. found that *Salmonella* can convert the immune suppressive MDSC into TNF-α-secreting cells with characteristics of neutrophils, which accompanied a loss of T_reg_ cells (60). Finally, Kim et al. reported that *Salmonella* recruits M1-like macrophages and reduces T_reg_ numbers in the tumor tissues, significantly elevating the proportion of M1/M2 macrophages (80).

### Therapeutic Combination

Despite its advantages as biotherapeutic, *Salmonella* in clinical trials did not show a significant effect as it did in animal experiments (8). The immunomodulatory effects of *Salmonella* suggest that it may work other therapeutics for achieving optimal tumor inhibition and overcome clinical barriers associated with monotherapies.

Several studies indicated that the combination of *Salmonella* with chemotherapeutics (such as 5-fluorouracil, cisplatin, cyclophosphamide, doxorubicin and *etc*.) causes a remarkably stronger tumor suppression (85–88). In a study of B-cell non-Hodgkin lymphoma, mice treated with the *Salmonella*/CHOP (cyclophosphamide, doxorubicin, vincristine, and prednisone) combination significantly delayed tumor growth and prolonged the animal survival compared to monotherapy. *Salmonella* also improved the side effects caused by chemotherapeutics (86). A similar conclusion was arrived from a study that used the combination of *Salmonella* and doxorubicin against breast cancer (85). Jia et al. (89) demonstrated that *Salmonella* strain VNP20009 improves the maximum tolerated dose and low-dose metronomic regimens of cyclophosphamide in a murine melanoma model. The VNP20009/cyclophosphamide combination reduced the circulating levels of vascular endothelial growth factor and tumor micro-vessel density compared to the monotherapy. Interestingly, tumor accumulation of *Salmonella* is also increased with the combination therapy than of *Salmonella* alone (89). Likewise, Triptolide and VNP20009 combination also strongly inhibited tumor angiogenesis and increased host immune response, which accompanied a decreased VEGF and neutrophils infiltration (42). Din et al. engineered *Salmonella* to lyse synchronously at a threshold population density and release encoded drugs, which exerted a better antitumor effect when combined with 5-fluorouracil (87). *Salmonella* also exerts better antitumor effects when combined with other drugs such as caffeine, valproic acid, gemcitabine, Chinese medicine herbal mixture and (90–94). As to the mechanisms, current studies suggest several possibilities. *Salmonella* could enhance gap junctional intercellular communication (GJIC) by increasing the expression of Cx43 (75, 95, 96), for promoting antigen presentation by DCs (75). Chang et al. (2013). suggest *Salmonella* enhanced the chemosensitivity of tumors to cisplatin, in which Cx43 was knocked down (95). A study with gemcitabine indicates that *Salmonella* strain A1-R pushes cancer cells from a chemo-resistant G_0_/G_1_ phase to the chemo-sensitive S phase (97). The same mechanism also appears to operate when cisplatin and paclitaxel with strain A1-R are combined (98, 99). *Salmonella* could also decrease the expression of P-glycoprotein (P-gp), a plasma membrane multidrug resistance protein, and its drug-efflux capabilities. This activity enhances the antitumor effects of 5-fluorouracil (96). P-gp down-regulation caused a decline of p-AKT, p-mTOR and p-p70s6K levels in tumors. The P-gp loss caused by *Salmonella* was rescued by recovering p-AKT, highlighting the important role of AKT/mTOR pathway (41, 52, 54, 96). Another study from Mercado-Lubo et al. (2016) indicated that P-gp loss by *Salmonella* is dependent its type III secretion effector, SipA. This process appears to require caspase-3 (100).

In addition to chemotherapy, *Salmonella* also enhances the antitumor effects of other therapeutics. Similar to *Bifidobacterium* and engineered *Escherichia* (101, 102), *Salmonella* synergistically augments radiation induced tumor growth inhibition (103–105). Murakami et al. (2015). used *Salmonella* strain A1-R as an adjuvant treatment after resection of metastases. This improved disease-free survival and lowered tumor burden in mice (106). Studies on the combination with nanomaterials further exert the tumor targeting advantages of *Salmonella (*107, 108). As a vehicle, *Salmonella* loads gold nanoparticles to the central tumor hypoxic regions, a restricted area for gold nanoparticles, and eventually enhances efficacy of radiation therapy (107). Another study combining with photothermal therapy also produced a promising result. Chen et al. (108). coated *Salmonella* strain VNP20009 with polydopamine *via* oxidation and self-polymerization. After systematic injection of coated *Salmonella*, near-infrared laser irradiation induced heating of polydopamine successfully restrained tumors in the targeted hypoxic regions (108). With the potential benefits of tumor immunotherapy, combining Salmonella with adoptive T cell therapy may yield a better antitumor effect, in which *Salmonella* sensitizes tumors to adoptive T cells therapy prevents tumor relapse and eventually eradicates tumors (109). Binder et al. reported that combining *Salmonella* expressing tumor-specific antigen ovalbumin with anti-PD-L1 antibody rescues antitumor function of endogenous PD-1^+^CD8^+^ T cells against long-established melanoma in mice (110).

### Clinical Trials

Since the first report in 1893 that patients infected by bacteria shows hemorrhagic necrosis of solid tumors (15, 16), many bacteria capable of colonizing and restraining tumors have been described (12). *Salmonella* was first shown to have a tumor therapeutic effect in animal models in 1935 (4). Until now several clinical studies on *Salmonella* have been reported (**Table 1**). As mentioned above, LPS and TNF-α are important for the therapeutic efficiency of *Salmonella* against tumors (45). Clinical trials with LPS from *Salmonella* in cancer patients were carried out since 1991 (111, 112). Engelhardt et al. (1991) intravenously administered escalating doses (from 0.15 ng/kg to 5.0 ng/kg) of purified-LPS from *Salmonella abortus equi* to cancer patients at 2-week intervals, and observed a strong rise in TNF-α, IL-6 and granulocyte-colony-stimulating factor (G-CSF) levels in serum. Two patients with colorectal cancer showed moderate antitumor activity in this trial (111). A Phase II trial of LPS was aimed at treating patients of colorectal and non-small cell lung cancer. Although LPS showed limited antitumor effects in non-small cell lung cancer, 3 out of 27 patients of colorectal cancer was had objective responses. Two patients with partial remissions were maintained for 7 and 8 months. While One patient with complete tumor remission was maintained for more than 3 years (112). However, the poor tumor response and toxicity limited the further application of purified LPS against tumors. Goto et al. (1996) reported that intradermal administration of LPS improves biological response (113). They showed higher tolerable doses of LPS produced more continuous induction of cytokines than previous intravenous administration. By combining LPS with cyclophosphamide, three out of five patients (ovarian cancer, cervical cancer and brain tumor) showed a significant response (113). A phase I trial using VNP20009 as therapeutic against tumors was first reported in 2002, which showed the safety of intravenous bolus infusions containing 10^6^ to 10^9^ CFU. Despite the high tolerance and colonization in some tumors, VNP20009 didn’t show objective response in patients against metastatic melanoma (114). Another clinical study using VNP20009 to treat multiple tumors in dogs was also carried out, in which 35 out of 41 animals were evaluable for antitumor response (116). A pilot trial further supported tumor colonization and the principle that *Salmonella* can be used as a delivery vehicle expressing exogenous growth inhibitory gene products into tumors. In this study, TAPET-CD, an attenuated *Salmonella* expressing the *E. coli* cytosine deaminase, successfully expressed the gene in 2 of 3 patients after intratumoral injection (115). Later trials used oral route for administering Salmonella. The attenuated *Salmonella* strain VXM01 was used in a phase I trial against advanced pancreatic cancer (120). VXM01 carries an VERFR-2 expressing plasmid, which aroused VEGFR2 specific T effector response and significant reduction of tumor perfusion, indicating anti-angiogenic activity in patients. These researchers further evaluated the safety of *Salmonella* in monthly boosting experiments and found a high VERFR-2 specific T cell response. Although patients received VXM01 showed adverse events such as lymphocyte reduction in blood, neutrophils increase and diarrhea, compared with placebo treatment, VXM01 demonstrated safety and efficiency on arousing VERFR-2 specific T cell responses (118). SalpIL2, a *Salmonella* strain carrying IL-2 expressing plasmid, was reported in a phase I clinical study with amputation and adjuvant doxorubicin for canine appendicular osteosarcoma. The disease-free interval (DFI) of dogs administrated with SalpIL2 was significantly prolonged than doxorubicin alone. Interestingly, the lower dose group showed longer DFI than group treated with highest SalpIL2 (117). However, another phase I trial using IL-2 expressing *Salmonella* strain, Saltikva, for metastatic gastrointestinal cancer patients showed no significant benefits. Saltikva increased circulating NK cells and NK-T cells, which promises the possibility for a multiple therapeutic strategy using *Salmonella* and other tumor immunotherapeutics (119). Above all, clinical trials of *Salmonella* strains against tumors are still in progress (121), which are likely provide a new generation of antitumor drugs through further modifications.

**Table 1:** Salmonella related clinical trials.

Clinical Trial Phase	Years	Strains	Administration	Major founding	Target Cancers	references
Phase I	1991	LPS (From Salmonella abortus equi)	Intravenously	Induce TNF-α, IL-6, and G-CSF moderate antitumor activity	Different	(111)
Phase II	1996	LPS (From Salmonella abortus equi)	Intravenously	1 complete remission, 2 partial remission from colorectal cancer patients	Colorectal and non-small cell lung cancer	(112)
	1996	LPS (Salmonella abortus equi)	Intradermally, with cyclophosphamide	Less toxic and more continuous cytokines induction than intravenous administration	Different	(113)
Phase I	2002	VNP20009	Intravenously	Safely for administration and tumor colonization for patients, induce TNF-α, IL-6, IL-1β, IL-12	Metastatic melanoma	(114)
Pilot trial	2003	TAPET-CD (Expresses cytosine deaminase)	Intratumorally	Capacity as gene delivery vehicle of Salmonella	Different	(115)
Phase I	2005	VNP20009	Intravenously	Demonstrated anti-tumor response in dogs	Different Canine tumors	(116)
Phase I	2016	SalpIL2 (Expresses IL-2)	Orally, with amputation and adjuvant doxorubicin	Prolong disease-free interval	Canine osteosarcoma	(117)
Phase I	2018	VXM01 (Expresses VEGFR2)	Orally	Reduction of tumor perfusion	Advanced pancreatic cancer	(118)
Phase I	2020	Saltikva (Expresses IL-2)	Orally	Increase circulating NK cells and NK-T cells	Metastatic gastrointestinal cancer	(119)

## Cancer Immunotherapy and Adverse Effects

Human immune system can recognize and eliminate foreign substances through innate and adaptive immunity to ensure the normal physiological function. Tumor cells have developed their specificity in many ways compared to normal cells, including biochemical composition, antigenic structures, and biologic behaviors (122, 123). These properties make it possible for immune system to recognize and eliminate tumor cells. However, the heterogeneity of tumors poses new challenges, such as differences between tumors of different patients, and in tumors of the same patient, and their individual surrounding environments (124). In addition, considering that tumor cells come from the body itself, they can use or enhance the mechanisms of the body’s autoimmune tolerance to evade the immune system in many ways, thus evading growth arrest. In the battle between host immune system and tumor cells, normal and strong autoimmune system can remove abnormal cells in time to ensure body’s normal physiological function. But, when tumor cells gained the ability to escape, host immune system cannot clear them. Therefore, tumor immunotherapy aimed at enhancing body’s antitumor immunity or reducing the immune evasion of tumor cells, will enable the host to tilt such imbalance (125).

### Development of Cancer Immunotherapy

The advent of smallpox vaccine in 1796 paved the way for subsequent immunogenic interventions (126). Dr William Coley, known as the “father of immunotherapy”, used “Coley’s toxins,” a mixture of live and inactivated *Streptococcus Pyogenes* and *Serratia Marcescens* to treat tumor patients, and found that this kind of toxins can be used to cope with various malignancies (15). Furthermore, a substantial relationship between tumorigenesis and immune system is revealed by the spontaneous regression of some patients with malignant tumor, and by the higher risk of cancer incidence in immunosuppressed populations (122). However, due to the limitation of therapeutic effect and poor understanding of therapeutic mechanism, as well as the development of chemo-, and radio-, therapies and surgery, immune therapeutic strategies to block tumors did not attract much attention in the following decades. After a long time, the effectiveness of tumor immunotherapy was established through the use of BCG against superficial bladder cancer (5). BCG can indirectly increase the expression of tumor antigens in tumor cells and extensively induce the expression of a large number of cytokines, thus enhancing the antitumor activity mediated by cytotoxic T lymphocytes, NK cells, neutrophils and macrophages (127). Besides, the possibility of TNF as an immunotherapy for tumors was suggested (128). Unfortunately, systematic treatment of TNF leads to severe toxicity, making it less likely to be used as a cancer therapeutic (129). Similarly, the USFDA approved IFN-α for treatment for hairy cell leukemia and IL-2 for metastatic kidney cancer and metastatic melanoma in 1986 and 1992 respectively, which have been shown to enhance the production of T cells in patients. However, subsequent clinical applications found the short duration of IFN-α in patients, and IL-2 could cause emergent therapeutic toxicity, which led to their gradual withdrawal from tumor therapy (11, 122). Sipuleucel-T (Provenge) is the first therapeutic autologous vaccine approved by the USFDA in 2010, which is used to treat symptomatic castration-resistant metastatic prostate cancer (122). The patient’s own DC cells were incubated *in vitro* with PA2024, a fusion protein constructed by prostatic acid phosphatase (PAP) and granulocyte macrophage colony stimulating factor (GM-CSF), and then re-infused back. Although Sipuleucel-T failed to prevent tumor progression, it extended survival time by an average of 4 months (130). Unlike modification of DC cells by Sipuleucel-T, Adoptive T cell therapy (including TIL, TCR-T and CAR-T) involves the isolation, modification and amplification of tumor-specific T cells *in vitro*, which are then infused back into patients to fight tumor cells, which has shown promising results in clinical trials for both hematologic and solid tumors (131). Among them, CAR-T was approved by the USFDA for relapsed and refractory B-cell acute lymphoblastic leukemia in pediatric and young adult patients (122). However, the death risk from cytokine storms and brain edema associated with treatment cannot be ignored at the same time (122). In addition, although adoptive cell therapy can achieve objective therapeutic effects, financial cost is another factor that needs to be controlled in the process of practical clinical promotion. For example, Sipuleucel-T requires about US $ 93,000 per one treatment period from collecting patients’ DC cells, transferring cells to laboratory for cultivation and modification *in vitro*, to reinfusing cells to patients. Its expensiveness undoubtedly limits the benefits of this kind of strategy for a large number of patients (132). In addition to adoptive cell therapy, as another pillar of today’s tumor immunotherapy, the development of immune checkpoint blockade opened a new door for tumor treatment. Persistent inflammation damages a body. To control the negative effects of excessive immune responses, many mechanisms limit them and control the degree of inflammation. The immune checkpoint is one such important feedback regulatory mechanism. Through a blockade of immune checkpoint protein, this strategy inhibits intrinsic down-regulation of immunity and mainly sustains the immune activity of T cells against tumor cells (133). In 2011, the first immune checkpoint drug anti-CTLA4 was approved by the USFDA for the treatment of metastatic melanoma (134). Subsequently, monoclonal antibodies capable of blocking PD-1 and PD-L1 have been successively developed for many types of cancers (133) (**Table 2**). In addition, drugs targeting other immune checkpoints are being continuously researched and developed (135). Oncolytic virus therapy is another promising approach for cancer immunotherapy. The understanding of viral therapy for tumors first emerged in the early 20th century, where tumor recession was noticed in patients who acquired viral diseases. The treatment of cervical cancer with rabies virus also suggests the possibility oncolytic viruses as a therapeutic (136). Viruses used for such therapies have been genetically attenuated to prevent their virulence to normal cells, while remaining tumor targeting and lysing capacities. These oncolytic viruses can carry antitumor agents into TME, which further enhances effectiveness (137). In 2015, the USFDA approved the first oncolytic virus antitumor drug, Talimogene Laherparepvec for melanoma (137). Currently, other clinical trials are being carried out for targeting a variety of different tumors, which are likely to provide more avenues for the expansion of tumor immunotherapy.

**Table 2:** Approved immune checkpoint inhibitors.

Agents	Type	Company	Approved Year	Indications
Ipilimumab	CTLA-4 mAb	Bristol-Myers Squibb	2011	Melanoma, liver cancer, cellular cancer, colorectal cancer
Pembrolizumab	PD-1 mAb	Merck	2014	Melanoma, lung cancer, head and neck squamous cell carcinoma, Hodgkin’s lymphoma, urothelial carcinoma, esophageal carcinoma, liver cancer, stomach cancer, kidney cancer, cervical cancer, primary mediastinal large B-cell lymphoma, Merckle cell carcinoma
Nivolumab	PD-1 mAb	Bristol-Myers Squibb	2015	Melanoma, lung cancer, head and neck squamous cell carcinoma, Hodgkin’s lymphoma, urothelial carcinoma, liver cancer, kidney cancer, colorectal cancer
Cemiplimab	PD-1 mAb	Sanofi SA/Regeneron	2018	Cutaneous squamous cell carcinoma
Atezolizumab	PD-L1 mAb	Genentech	2016	Urothelial carcinoma, lung cancer
Avelumab	PD-L1 mAb	Merck Serono	2017	Urothelial carcinoma, renal carcinoma, highly malignant skin cancer
Durvalumab	PD-L1 mAb	Medimmune	2017	Urothelial carcinoma

### Following Adverse Events

In the scope of cancer immunotherapy, immune checkpoint blockade can effectively activate tumor-specific T cells and provokes the development of antitumor microenvironment. Immune checkpoint, CTLA-4 and its ligands B7-1 and B7-2 are mainly concentrated in human and mouse secondary lymphocyte organs, while PD-1 is more widely expressed in non-lymphocyte T cells, and its ligand PD-L1 is also more widely expressed in peripheral inflammatory tissue sites, making it a key factor in regulating peripheral effecter T cell responses (138, 139). Accordingly, PD-1 plays a critical role in regulating CD8+T cell exhaustion than CTLA-4, which is better at inactivating and initiating T cells (140–142). In addition, CTLA-4 and PD-1 are also widely found in B cells, NK cells, DC cells, monocytes, granulocytes, NKT cells and other immune-related cells (138, 139). Therefore, the effects of immune checkpoints on immune system are extensive, which is bound to involve a number of complex mechanisms requiring further research in the future. The immune system recognizes not only exogenous pathogens, but also some antigens in normal tissue cells. Therefore, immune tolerance, as a protective measure, prevents the pathogenic autoimmune responses caused by the excessive immune to native tissues, symbiotic organisms and harmless environmental antigens. These responses include allergic diseases (such as urticaria, bronchial asthma and anaphylactic shock) and other autoimmune diseases might damage host (143). However, immune checkpoint blockade might increase the risk of off-tumor inflammatory responses and disturb the immune tolerance regulation in patients (143). Clinical studies have shown that some patients who received immune checkpoint blockade, developed immune-related adverse events (irAEs). In some cases, it was life-threatening (144). The irAEs caused by immune checkpoint blockade are varied, such as rash, colitis, hepatotoxicity, pneumonia, and pituitary inflammation. More seriously, irAEs could cause permanent damage to some organs, such as insulin and adrenal corticosteroid deficiency (145–148). In addition, block different immune checkpoint are associated with different types of irAEs. For example, patients who received CTLA-4 blockade were more likely to experience pituitary inflammation and colitis, while those treated with PD-1 antibodies were more likely to develop thyroid dysfunction (147). Current statistics show that PD-L1 blockade has the lowest probability (12-24%), while CTLA-4 blockade has the highest probability (60-85%) of producing irAEs. Combined blockade of both PD-1 and CTLA-4 resulted in more serious adverse reactions (143). Patients with preexisting autoimmune diseases are often excluded from checkpoint blockade therapy. However, clinical analyses show that not all preexisting autoimmune diseases patients had an aggravating autoimmune disease after immune checkpoint blockade (149, 150). In a study of Ipilimumab in cancer patients with different preexisting autoimmune diseases (6 with rheumatoid arthritis, 5 with psoriasis, 6 with inflammatory bowel disease, 2 with systemic lupus erythematosus, 2 with multiple sclerosis, 2 with autoimmune thyroiditis, and 7 with other conditions), only eight from them developed worsening autoimmune diseases, while cortisol intervention effectively inhibited such adverse reactions (149). Similarly, another retrospective study exploring the impact of a PD-1 inhibitor on preexisting autoimmune disease patients showed that only 52 of 119 patients had autoimmune disease progression. Patients also experienced relative relief of adverse reactions after receiving the intervention of corticosteroids and steroid-sparing agents (150). It is not yet understood under what circumstances the incidence of irAEs will increase, which autoimmune diseases are more likely to be induced by immune checkpoint inhibitors, and whether different autoimmune diseases require different defenses to induce them. With the widespread use of checkpoint blockade drugs, we are likely to see more unforeseen adverse conditions in preclinical studies. Therefore, how to determine the maximum dose to balance the negative effects from adverse events in patients, and how to reduce the adverse events caused by immune checkpoint blockade is an important direction for the development of tumor immunotherapy in the future.

Currently, the possible mechanisms of irAEs include the following aspects: 1. enhance autoreactive T and B cells, or inhibit the activity of T_reg_ cells to induce the preexisting subclinical autoimmune condition; 2. Directly trigger new autoimmunity or inflammation; 3. Disturb the balance of gastrointestinal symbiotic bacteria and causes gastrointestinal disruption; 4. Damage normal tissues around tumor tissues, or directly affect tissues with high expression of immune checkpoint, such as hypophysis (143). Therefore, one of the key issues that needs to be addressed in immunosuppressive therapy is how to isolate irAEs-related adverse inflammation from tumor immunity enhanced by immune checkpoint blockade, which might be influenced by drug toxicity, tumor types, and different type of irAEs diseases (143). Treatment with CAR-T therapy also resulted in irAEs in some cancer patients. The combination anti-IL-6 receptor can effectively inhibit it during the CAR-T therapy (151).

However, unlike immune checkpoint blockade, the irAEs induced by CAR-T were mainly attributed to the IL-6-induced cytokine storm. In contrast, possible mechanisms of irAEs discussed above, and the causes of irAEs caused by immune checkpoint blockade are more extensive.

## Integration of Salmonella Into Combination Immunotherapy

Cancer immunotherapy has made remarkable achievements and become another important antitumor strategy after chemoradiotherapy and surgery. However, similar to other therapies, in addition to the excellent antitumor effects, many side effects and limitations have been observed in some patients who received immunotherapies. For example, some patients with cancer immunotherapy were found to develop autoimmune reactions, cytokine release syndrome and vascular leak syndrome. Some severe cases are even fatal for patients (11). Patients with preexisting autoimmune disorders are often excluded from the treatment for immune checkpoint blockade (149). A rational integration of other approaches is expected to enhance the antitumor therapeutic effects and reduce immune-related side effects caused by cancer immunotherapy. In recent years, some studies showed that, in addition to their own antitumor toxicity, oncolytic virus can also serve as engineering platforms for immunotherapy (152). Engineered oncolytic virus were shown selectively replicate, destroy tumor tissues and enhance host antitumor immunity (137). The USFDA has approved an oncolytic antitumor drug “Talimogene laherparepvec (Imlygic)” in 2015, which is derived from herpes simplex virus type 1 and indicated for patients with melanoma recurrent after initial surgery (11). Similarly, the antitumor effects of bacteria were also being explored widely (12). For example, *Salmonella* has been used as promising antitumor agent and deliver cytotoxic agents for its ability in tumors targeting, cytotoxicity, reversal of immune tolerance (8). Therefore, a combination of *Salmonella* with other immunotherapeutic could further exert its anticancer effects and reduce the therapeutic resistance and side effects during current cancer immunotherapy (**Table 3**).

**Table 3:** Integration of *Salmonella* into current immunotherapies.

Co-administer *Salmonella* with Current Immunotherapeutics	Engineered Salmonella Mediated Therapy	Carrying/Secreting RNAi or Antibodies of Immune Checkpoints
•Induces antitumor cytokines in TME	•Enhances tumor antigen(s) expression	•Inhibits PD-L1 of cancer cells
•Recruits more anti-tumor immune cells into the TME	•As a gene therapeutic delivery to affect cancer-related genes	•Activates CTL in the TME
•Attenuates immunosuppression in the TME		•Enhances targeting ability of immune checkpoint therapy and weakens irAEs

### Delivery for Antitumor Immunomodulators

With the development of drug delivery systems and higher targeting requirement for antitumor drugs, an increasing number of studies are employing nanoparticles, scaffolds and hydrogels to improve the efficacy and safety of cancer immunotherapy (11). Because of tumor targeting, intrinsic oncolytic and host immune response augmenting abilities, *Salmonella* has been widely studied as a drug carrier for oncotherapy. Gene-editing techniques enable tumor-targeting *Salmonella* to interfere genes expression in tumor cells or macrophages, further suppressing the tumor growth. It also enables *Salmonella* to directly express (or secrete) tumor antigens, cytokines, antibodies and *etc*., which affects TME and the antitumor ability of host immune system (8).

Many genes that promote tumor development have been found. Mutations in some of them are directly associated with tumorigenesis. Engineered *Salmonella*, as a kind of potential tumor gene therapy platform, could carry target genes into patients’ tumor cells, affecting the expression of cancer-related genes. For example, *TP53* is a widely mutated tumor suppressor in cancers, which prevents an elimination of damaged cells. Loss of the p53 protein is one of the reasons for resistance to chemotherapeutics (153). *Salmonella* carrying a eukaryotic expression wild-type p53 plasmid to colonize tumor sites restored its expression of in tumor cells effectively in inhibited tumor growth and regained the sensitivity to chemotherapeutics (154, 155). In addition, *Salmonella* carrying eukaryotic expression plasmids that express shRNAs which target oncogenic products like STAT3, Survivin, Bcl-2, and HIF-1 strongly suppressed tumor growth in xenograft models (156–159). The use of *Salmonella* to overexpress tumor antigens in cancer cells, such as Legumain, PCSA, AFP, *etc*., has also been shown to inhibit tumor growth effectively (160–162). Similarly, genetically modified *Salmonella* successfully induced tumor cells to secrete immune-related cytokines, such as IL-12, IL-4, and IL-18. These cytokines modify the host immune responses and inhibit tumor growth (163, 164). For *Salmonella* caring prokaryotic expression system, direct expression or secretion of target proteins might be more effective for influencing the TME. Studies on osteosarcoma treated with an IL-2 secreting *Salmonella* have shown the tumor repression effect in mice model (165). A pre-clinical study of Canine osteosarcoma treated with *Salmonella* expressing IL-2 has also shown a prolongation of disease-free interval (117). IL-2 secreting *Salmonella* increased circulating NK cells and NK-T cells in patients with metastatic gastrointestinal cancer (119). In addition to IL-2 (approved for metastatic kidney cancer and metastatic melanoma in 1992 and 1998), IFN-α has been approved by the USFDA for the treatment with hairy cell leukemia (1986) (11), and TNF (tasonermin) was licensed in Europe for irresectable soft tissue sarcoma in the 1999. However, due to the short effectiveness and serious adverse reactions, they are not used in clinical treatment nowadays (11, 122). Studies using IFN-γ or TNF-α secreting *Salmonella* have found significant inhibitory effects on the growth of melanoma in mice, demonstrating the possibility for further use of this type of therapy (166, 167). Flagellin, also exhibited strong antitumor effects (43, 44). *Salmonella* secreting flagellin B inhibits tumor growth by regulating TME *via* TLR5 and shifts macrophages from M2-like suppressive activities to M1 phenotypes in the microenvironment. Besides, flagellin diminished the numbers of T_reg_ cells in the TME (46, 47). Binder et al. (2016). constructed an antigen (SIINF) producing *Salmonella*, which satisfied the high-antigen expression requirement of adoptively transferred T cells and prevented tumor relapse of a fibrosarcoma which has low antigen expression (109). In addition, genetic programming may further enhance the delivery ability of *Salmonella*. Sreyan *et al*. (2019) engineered a quorum-sensing *E. coli* to specifically lyse upon tumor accumulation (168). Based on the tumor-targeting ability, quorum sensing lysis was a promising strategy to enhance *Salmonella* releasing anti-tumor agents.

### Combination With Immune Checkpoint Blockade

The complete activation of T cells requires two different signals. One is the contact of antigens and major histocompatibility complex (MHC), respectively on the cell surface of antigen presenting cells (APCs) with the T cell receptor, which produce antigen specific T-cells. The others are antigen independent co-signaling molecules, including co-stimulators and co-inhibitors, which are also known as immune checkpoint (134). The presence of immune checkpoint is conducive to controlling and regulating the persistence of the immune response and preventing the pathogenic autoimmune responses (143). The blockade of immune checkpoint permits the killing of tumor cells by unleashing killer T cells. Since the first CTLA-4 block drug, Ipilimumab, was approved for metastatic melanoma in 2011 (134), immune checkpoint blockade has shown impressive durable responses and therapeutic effects, elevating the development of cancer immunotherapy to a new level. However, as described in the above, the irAEs generated due to immune checkpoint blockade pose a potential hazard for the long-term clinical use of this therapy. The fact that only some patients benefit from such treatment adds to the barriers to its wider use (169). In terms of blocking PD-1/PD-L1, the infiltration of T cells into tumors and the expression of PD-L1 will largely affect the therapeutic effect of immune checkpoint inhibitors. However, clinical studies also found that some PD-L1 positive patients had poor feedback on immune checkpoint blockade, while others patients with negative PD-L1 were also able to respond (169), which provides a new requirement for further understanding the role of immune checkpoint in tumor immunotherapy to improve this strategy.

PD-L1 expression in tumor tissues can be induced by IFN-γ, secreted by tumor-specific T cells after recognizing tumor cells. The up-regulation of PD-L1 is not only limited to tumor cells in the TME (170–172). This physiological induction of PD-L1 leads to the escape of tumor cells from T cell response and immune tolerance (169). Theoretically, for patients with high PD-L1 expression, blocking PD-1/PD-L1 should be able to eliminate the immune tolerance and achieve the therapeutic purpose. However, clinical data analyses showed that some patients lacked T cell infiltration could still not be effectively treated, although the expression of PD-L1 has been down-regulated (169). In fact, most patients with no response to PD-1/PD-L1 blockade show little T cell infiltration and negative PD-L1 expression (170, 172). However, clinical trials in melanoma have shown that the combination of Nivolumab (αPD-1) and Ipilimumab (αCTLA-4) can restore the therapeutic effects of Nivolumab and extend progression-free survival in patients with negative PD-L1, compared with those treated with Nivolumab alone (173, 174). The enhanced effect of PD-1/PD-L1 blockade is attributed to CTLA-4 inhibition by Ipilimumab, which lead to a marked diversification of the peripheral TCR repertoire and increased infiltration of T cells into tumor tissue (174). Interestingly, T cell infiltration increased by anti-CTLA-4, could also cause PD-L1 up-regulation through IFN-γ induction (169). The effect of Nivolumab in the combined treatment group may offset the negative effect of Ipilimumab on inducing PD-L1 after increasing T cell tumor invasion.

Currently, the success of *Salmonella* in animal studies has not been fully replicated in clinical studies (8). The reasons for such weaker response are manifold, such as the biological differences between human and animals, optimization of bacterial administration (routes, numbers, genetic modifications) and *etc*. Among them, the relationship between *Salmonella* and immune checkpoint has not received sufficient attention. Chen et al. found the down-regulation of PD-L1 expression in a variety of mouse and human tumor cells treated with *Salmonella*, affects apoptosis of co-cultured PD-1-expression cells (79). However, PD-L1 is also expressed in other non-tumor cells in TME, such as stromal cells, myeloid-derived cells and infiltrating immune cells (169, 175). Among them, a study on colon cancer found that PD-L1 expressed by mesenchymal stromal cells could decrease T cells proliferation, activation, and promote tumor growth (175). Notably, in addition to tumor cells, many studies have found that *Salmonella* induces the expression of PD-L1 in different cell types. *Salmonella* induces PD-L1 in B cells, which might use this mechanism to evade cytotoxic effector response and enable B cells as a reservoir for the bacteria (176). Further studies showed that the up-regulated PD-L1 in B cells (induced by *Salmonella)* impaired T cell response (177). Similarly, *Salmonella* also induces the expression of PD-L1 in DCs and CD4^+^ T cells (178, 179). The PD-L1 up-regulation is associated with the Pathogenicity Island2 of *Salmonella* (180). The above confounding observations raise, whether the PD-1/PD-L1 pathway positive or negative role in *Salmonella* mediated tumor therapy. Recently, many studies on the combination of *Salmonella* and immune checkpoint blockade were reported (110, 181–183). Binder et al. (2013) constructed an antigen-producing *Salmonella* against long-established immunogenic melanomas. It is worth noting that, 80% tumor rejection and expansion of tumor-specific CD8^+^ T cells were observed after the treatment combining antigen (ovalbumin) producing *Salmonella* with anti-PD-L1 antibody (110). Besides, in two other studies focusing on *Salmonella* carrying PD-1 siRNA, Zhao et al. indicated a promising antitumor strategy that integrating immune checkpoint blockade into *Salmonella* (182, 183). However, a study on combining anti-PD-1 antibody with *Salmonella* carrying IDO siRNA against colorectal tumors showed no additional tumor growth inhibition than group of *Salmonella* alone (181), suggesting that the synergistic antitumor effect of *Salmonella* and immune checkpoint inhibiting needs to be further explored.

## Conclusions and Future Perspectives

In recent years, the success of immune checkpoint blockade and CAR-T have established immunotherapy as a major cancer treatment (10). In addition, BCG, oncolytic virus and other immune-related remedies are approved for the treatment of certain tumors (5, 137, 152). The anti-tumor ability of cancer immunotherapy involves many factors, including the expression of tumor antigens, infiltration of effective immune cells into TME, levels of inflammatory factors and immunosuppression signals (125, 126, 132, 135). Current clinical reports also indicate the limitations of single agent immunotherapy and the more effective combinations are needed for augmenting immunotherapies (140, 141). Besides, the non-tumor-related immune effects of cancer immunotherapy also lead to a large number of adverse reactions, which puts forward higher requirements for the further development and application of cancer immunotherapy (143). The rapid development of *Salmonella* has demonstrated its potential as a novel immunotherapy for cancer. Although reported clinical trials have not yielded exciting results (8), the proven tumor targeting, immune regulating ability and engineering plasticity indicate that *Salmonella* still has great potential for further development. For example: 1. *Salmonella* can colonize and grow in tumors. If we take advantage of this and combine with other therapeutics may help reduce the frequency of treatment and prolong the effectiveness of a single treatment; 2. Although the elimination of its toxicity significantly weakens the intrinsic oncolytic activity of *Salmonella*, the use of biochemical techniques to restore or even enhance oncolytic activity may be a solution to the poor clinical efficacy of *Salmonella*; 3. *Salmonella* has been shown to enhance the sensitivity of tumors to chemotherapy in several ways. Future research should focus on adjuvant and combination therapies; 4. The development of tracer method of *Salmonella* is expected to provide an *in vivo* detection of tumor sites and metastasis; 5. Integrating the tumor targeting of *Salmonella* is expected to enhance the tumor specificity of PD-1/PD-L1 blockade, and reduce non-tumor related immune regulation. The enhancement of tumor specificity is expected to minimize a series of clinical adverse effects caused by immune checkpoint blockade; 6. The induction of immune cell infiltration by *Salmonella* in TME may improve the poor response to cancer immunotherapy in some patients with low immune cell infiltration. It may also convert immunosuppressive signals in TME of some patients; 7. The plasticity of *Salmonella* as attenuated engineered bacteria make it promising tool for multiple anti-tumor strategies simultaneously. For example, the advantages of combining PD-1 and CTLA-4 antibody may be achieved through engineered-*Salmonella* secreting both of them simultaneously.

Although the cancer treatment of *Salmonella* holds great promise, we cannot ignore the problems that need to be solved in the future therapies. These include comprehensive consideration of clinical safety, optimization of dose and interval of administration, timely elimination of bacteria after administration, concerns about antibiotic use, and quality assurance of mass production of live bacteria *etc* (8). However, more research of *Salmonella* is needed to further explore the modification and treatment strategies. In the context of clinical limitations and side effects of current cancer immunotherapy, the tumor-homing ability and influence on host immune system raises the possibility for the intervention with *Salmonella*. It is expected that bacterial treatment against cancer will likely flourish in the near future, expanding the frontiers of cancer immunotherapy.

## Author Contributions

DW and XW designed and wrote this manuscript. DK, BG, and LZ provided critical comments, concepts, and insights. All authors contributed to the article and approved the submitted version.

## Funding

LZ is supported by the National Natural Science Foundation of China (grant numbers 81773217), Research Fund of Jilin Provincial Science and Technology Department (grant numbers 20200404120YY and 20190701065GH), Jilin Province Health Technology Innovation Project (grant numbers 2019J030), the Fundamental Research Funds for the Central Universities, JLU and Chunhui international research project of Ministry of Education. DK is supported by the Cigarette Restitution Fund of the University of Maryland Greenebaum Cancer Center.

## Conflict of Interest

The authors declare that the research was conducted in the absence of any commercial or financial relationships that could be construed as a potential conflict of interest.

## Abbreviations

CAR-T, chimeric antigen receptor T cell therapies; BCG, *Bacillus Calmette Guerin*; TME, tumor microenvironment; TNF, tumor necrosis factor; HIF, hypoxia inducible factor; VEGF, vascular endothelial growth factor; VEGFR, VEGF receptor; PAMPs, pathogen-associated molecular patterns; LPS, lipopolysaccharide; T_reg_, regulatory T cells; TLR, Toll-like receptor; NO, nitric oxide; iNOS, inducible NO synthase; MMP, matrix metalloproteinase; IFN, interferon; IL, interleukin; CXCL, chemokine ligand; MDSC, myeloid-derived suppressor cell; CSF, colony stimulating factor; MIP, macrophage inflammatory protein; NK, natural killer; ARG, arginase; TGF, transforming growth factor; NLRP3, NLR family pyrin domain containing 3; IPAF, interstitial pneumonia with autoimmune features; ATP, adenosine triphosphate; CTLs, cytotoxic T lymphocytes; DC, dendritic cell; Cx43, connexin 43; IDO, indoleamine 2, 3-dioxygenase; GJIC, gap junctional intercellular communication; DFI, Disease-free interval; PAP, prostatic acid phosphatase; TIL, tumor infiltrating lymphocyte; TCR, T cell receptor; TCR-T, TCR T cell therapies; irAEs, immune-related adverse events; MHC, major histocompatibility complex; APC, antigen presenting cell.
